# Clinical advantages of suctioning flexible ureteroscopy with intelligent pressure control on treating large upper urinary tract calculi

**DOI:** 10.3389/fsurg.2025.1554964

**Published:** 2025-07-02

**Authors:** Weiping Cai, Bin Zheng, Xinwei Li, Xingjian Gao, Zedong Zhang, Yijin Lu, Hualong Zhao, Junhong You, Gangfeng Zheng, Weilong Bao, Yutong Lai, Yisong Lv

**Affiliations:** Department of Urology, The General Hospital of Fujian Energy Group, Fuzhou, Fujian, China

**Keywords:** Clavien-Dindo classification, stone-free rate, suctioning flexible ureteroscopy with intelligent pressure control, upper urinary tract calculi, calculi

## Abstract

Suctioning Flexible Ureteroscopy with Intelligent Pressure Control (SFUI) has the advantage of automatically capturing and breaking urinary tract calculi while maintaining renal pelvic pressure stability. This retrospective study aimed to evaluate the efficacy of SFUI in treating upper urinary tract calculi of large sizes. A total of 200 patients with upper urinary tract calculi who underwent SFUI treatment in a single location from 2020 to 2021 were included. Outcomes were a one-session stone-free rate (SFR), one-month SFR, and complications within 4 weeks after SFUI classified by Clavien-Dindo grades. Patients’ median age was 50.0 years and a majority (65%) was males. Among them, 119 patients had small calculi (<2 cm) and 81 patients had large calculi (≥ 2 cm); 1 (0.8%) patient in the small calculi group, and 4 (4.9%) patients in the large calculi group had complications ≥ Grade II. Multivariable analysis showed that the large calculi group had significantly lower odds ratio for total one-session SFR [adjusted odds ratio (aOR): 0.22, 95% confidence interval (CI): 0.07–0.67, *p* = 0.008, S-value = 6.97] and one-month SFR (aOR: 0.27, 95% CI: 0.09–0.83, *p* = 0.022, S-value = 5.64) compared to the small calculi group, whereas calculi size was not associated with complication rate (aOR: 2.62, 95% CI: 0.23–29.32, *p* = 0.43, S-value = 1.20). In conclusions, SFUI is safe and effective for immediate stone removal after surgery. The very low complication rate benefits fast recovery, especially for patients with large calculi.

## Introduction

Urinary tract calculi are primarily managed using minimally invasive methods such as ureteroscopy lithotripsy and percutaneous nephrolithotomy (PCNL), with the choice of treatment depending on patient factors, stone size, equipment, and expertise (1). Flexible ureteroscopy lithotripsy (FURL) is widely used for small renal stones due to its high stone-free rates (SFRs) and low complication rates (2–7). However, the high perfusion rate during FURL can lead to elevated renal pelvic pressure and backflow (8), which may result in complications like postoperative fever and systemic inflammatory response syndrome (9, 10).

As stone size increases, SFRs for FURL decrease significantly (2, 7). For renal stones ≥ 2 cm, the American Urological Association (AUA) and the European Association of Urology (EAU) recommend PCNL as the first-line treatment choice (11, 12). Despite the existing guidelines, controversy remains regarding the optimal treatment for larger stones, particularly those between 2 and 3 cm. While PCNL achieves a high SFR with effective immediate stone removal, it is associated with higher complication rates and longer hospital stays compared to FURL (4, 6, 7, 13, 14). FURL, though less invasive, is still questioned for its efficacy in larger stones, with limited high-quality evidence to support its use for this subset of patients. Previous meta-analyses documented that while FURL is generally safe and effective, it has significantly lower SFRs compared to PCNL for stones larger than 2 cm (15, 16). Thus, there is a need for treatment methods that achieve high SFRs while minimizing complications, especially for larger stones.

Suctioning Flexible Ureteroscopy with Intelligent Pressure Control (SFUI), a new variation of FURL, has demonstrated high lithotripsy efficacy and low complication rates (2%–5%) in patients with upper urinary tract calculi and a solitary kidney (17–19). However, current studies have not adequately explored this relatively newer technique. Therefore, there is a pressing need for further investigation to address the gaps in evidence regarding SFUI. This retrospective cohort study aimed to confirm the safety and effectiveness of SFUI in treating large calculi in different locations of the upper urinary tract, providing data to guide clinical decision-making and potentially bridge the gap between FURL and PCNL for larger stones.

## Methods

### Study design and patient selection criteria

This retrospective cohort study reviewed the medical records of patients who underwent SFUI surgery at the Department of Urology, General Hospital of Fujian Energy Group, China, between July 2020 and August 2021. Inclusion criteria were: (1) a diagnosis of urinary tract calculi; and (2) a comprehensive diagnostic work-up, including medical history, routine preoperative exams, and laboratory tests such as urinalysis, urine culture, blood tests, renal function assessment, and imaging studies. No specific exclusion criteria were applied. The study protocol was reviewed and approved by the Institutional Review Board of our institution, and informed consent was waived due to the retrospective nature of the study.

### Operative procedure

Before the operation, a thorough assessment of the patient's history of urinary stones will be conducted, including whether the stones are primary or recurrent and the presence of ureteral stenosis. Before the operation, CT plain scan with 3D reconstruction and x-rays were used to assess the size and volume of the stones. x-rays were used to confirm stone radiopacity, establish a baseline image, and assess whether the stone could be monitored postoperatively using x-ray, which involves lower radiation exposure.

**Figure 1:**
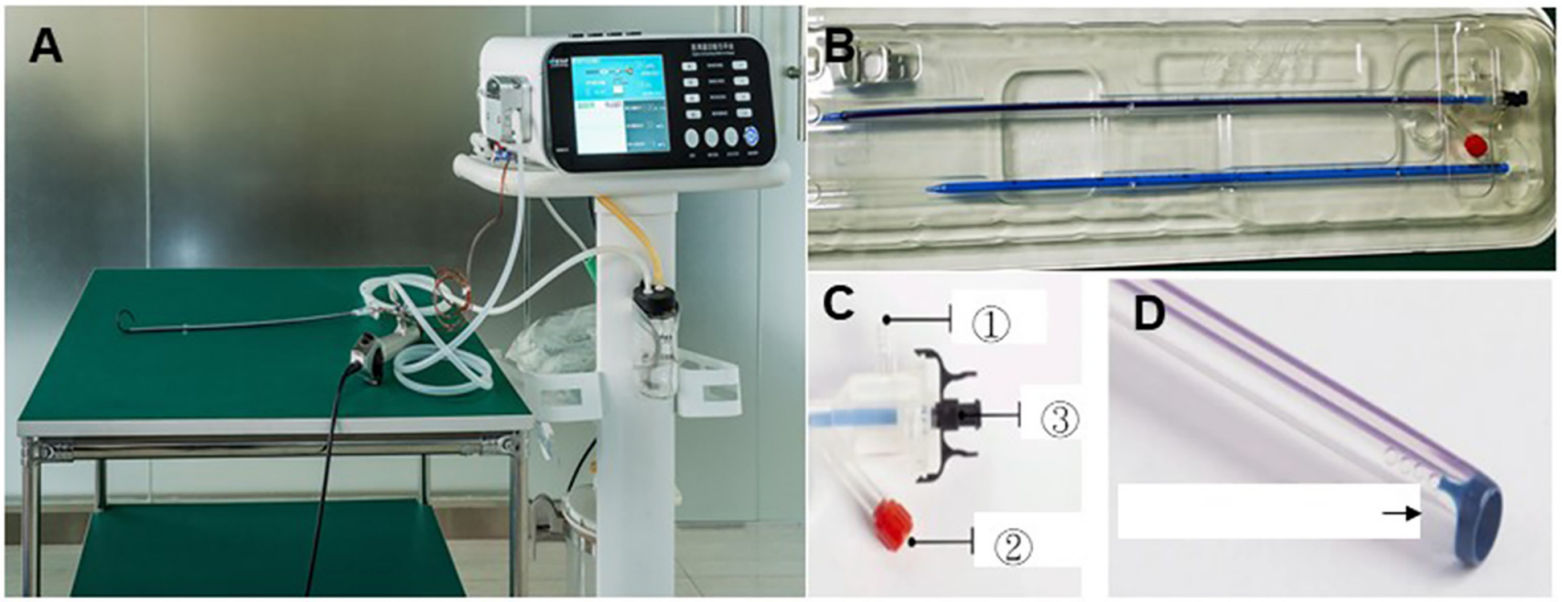
The SFUI system. **(A)** A patented irrigation and suction platform, consisting of a main control unit, an infusion device, a suctioning device, and a pressure feedback unit. The perfusion flow, pressure control value, and pressure limit value (30 mmHg) can be monitored on the main control unit during surgery. **(B)** A UAS with a transparent pressure-sensitive tip. The UAS has **(C)** one pressure sensor in the front end and **(D)** two connection channels at the back end, ① to vacuum device with suction effect and ② to pressure monitoring feedback device. ③ is the connecting channel to a flexible ureteroscope. UAS, ureteral access sheath.

All surgeries were performed by the same surgeon with the SFUI system. The SFUI system contained a patented irrigation and suctioning platform (Patent No. ZL201420055766.5) and ureteral access sheath (Patent No. 201420055134.9) (Figure 1A–D) (17–19). The ureteral access sheath (outer diameter: F14.9; diameter of the working channel: F12; length 35–45 cm) has a pressure-sensitive tip. The very advantage of the SFUI system is keeping renal pelvic pressure (RPP) stable during operation, which is achieved by precisely regulating infusion flow and controlling the vacuum suctioning through computerized real-time recording and monitoring of RPP with a pressure feedback system (17–19).

The whole operative procedure has been described previously (17–19). Briefly, patients were in the oblique supine lithotomy position with the diseased side upward under general anesthesia (Figure 1E). Ureteroscopy was performed with a semi-rigid 8/9.8F ureteroscope (Richard Wolf, Germany) with a flexible 0.032-inch guidewire (Innovel, China) inserted into the renal collecting system. The ureteral access sheath was inserted into the proximal ureter along the guidewire without fluoroscopic guidance, and then a disposable flexible ureteroscope (Pusen, China) was inserted into the sheath for a comprehensive inspection of the delivery location of the transparent sheath between the renal pelvis and ureter.

The pressure sensory and suctioning channels were connected to the irrigation and suctioning platform when the transparent sheath reached the targeted position. Perfusion flow was set at 50–150 ml/min. RPP control value was set at −15∼−5 mmHg. The upper-limit value was set at 30 mmHg. The stone was broken by a holmium laser (Raykeen, China) at 0.8–1.2 J/pulse with a frequency of 20–30 Hz (energy ranged from 16 to 36 Watts). During lithotripsy, the scope body was moved back and forth slightly to facilitate suctioning out the small gravel particles, while gravel particles larger than the sheath gap but smaller than the ureteral access sheath were suctioned out by withdrawing the scope intermittently without the need for stone basketing. For patients in whom the indwelling ureteral access sheath was not successful, a 7F Double-J ureteral stent (Asymchem Inc., China) was placed for 2 weeks to facilitate the UAS placement for flexible ureteroscopy. Considering that the mucosa of the ureter may be hurt by repeated suction of stone fragments, a 7F Double-J ureteral stent was placed at the end of the operation and remained indwelling for 4 weeks to protect the ureter. Patients were followed at 4 weeks after the operation.

Not all patients successfully had an introducer sheath placed on the first attempt. Approximately 13% required the placement of a double J tube for two weeks before undergoing surgery due to initial placement failure.

Postoperatively, routine color Doppler ultrasound examinations at 3 and 6 months will be conducted to evaluate changes in hydronephrosis and assess for any postoperative ureteral stenosis, ensuring timely intervention if needed.

### Clinical outcomes

The primary outcomes were the SFRs at one session and at one month after surgery. The secondary outcome was complications classified by Clavie-Dindo grade (20, 21) within 4 weeks after surgery. Stone size was determined by the maximal length shown in the KUB x-ray or CT image. For multiple stones, the sum of the maximal length of all stones was calculated. Stone-free was defined as no residual stone or left residual stone < 4 mm in size recognized by KUB x-ray images. Based on the Chinese Guideline for Diagnosis of Urology and Male Diseases 2019, intense follow-up is allowed for residual stones ≦4 mm without obstruction or infection.

### Statistical analysis

To compare different groups, categorical variables are presented as N (%) and performed by the Chi-square test or Fisher's exact test, as appropriate. When more than 20% of cells have expected frequencies < 5, we used Fisher's exact test because applying the approximation method is inadequate (22). The normality of continuous data was examined by the Shapiro–Wilk test. Continuous variables with normal distribution are presented as mean ± standard deviation (SD) and performed by Student's *t*-test; non-normally distributed data are presented as median (interquartile range, 25th-75th percentile, IQR) and performed by the Wilcoxon rank-sum test. Adjusted odds ratios (aOR) and 95% confidence intervals (CI) were calculated for outcomes adjusted for *p*-value < 0.15 in univariate analysis using multiple logistic regression analysis. *p*-value < 0.05 was considered statistically significant. We also provided the *S*-value (the Shannon information, surprisal, or self-information), which is a logarithmic transformation of the *P*-value: *S*-value = − log_2_ (*p*-value) for the logistic regression model. The 95% CI includes the range of values which are compatible with the data, that is, statistical testing of values provides no > 4.32 bits (*S*-value > 4.32) of information against them assuming the background assumptions are correct. All statistical analyses were performed using SAS 9.4 statistical software (SAS Institute, Inc., Cary, NC, USA).

## Results

### Characteristics of the patients

A total of 200 patients were included, of whom 119 patients had stones <2 cm and 81 had stones ≥2 cm. Table 1 shows patients' baseline demographic and clinical characteristics. Patients' median age was 50.0 years. Most patients were male (65.0%). Among all patients, the main location of stones was the ureter (40.0%), median/lower calyx (32.5%), and renal pelvis (15.5%). Patients with stones ≥2 cm had significantly higher proportions of a history of stones (39.5% vs. 23.5%, *p* = 0.02), hypertension (37.0% vs. 22.7%, *p* = 0.03), multiple stones (44.4% vs. 18.5%, *p* < 0.001) and stones >1000 HU on CT scan (43.2% vs. 18.5%, *p* < 0.001) compared to those with stones <2 cm. In the <2 cm stone group, stones were primarily located in the ureter (49.6%) and the middle/lower calyx (32.5%), whereas in the ≥2 cm stone group, stones were predominantly found in the middle/lower calyx (32.1%). No significant differences were found in age, sex, BMI, stone composition, or hydronephrosis between the two groups.

**Table 1:** Demographic and clinical characteristics of patients with stone size < 2 cm and ≥2 cm.

Characteristics	Total (*n* = 200)	Stone size (cm)		*p*-value
		<2 (*n* = 119)	≥2 (*n* = 81)	
Demography				
Age, years	50.0 (42.0–60.0)	50.0 (41.0–60.0)	53.5 (44.0–60.0)	0.21^c^
Sex				0.48^b^
Male	130 (65.0)	75 (63.0)	55 (67.9)	
Female	70 (35.0)	44 (37.0)	26 (32.1)	
Clinical characteristics				
BMI, kg/m^2^	24.8 (22.6–26.7)	24.8 (22.6–26.6)	24.8 (22.8–26.7)	0.62^c^
Stent in ureter	39 (19.5)	27 (22.7)	12 (14.8)	0.17^b^
Infection	153 (76.5)	87 (73.1)	66 (81.5)	0.17^b^
Comorbidity				
History of stones	60 (30.0)	28 (23.5)	32 (39.5)	0.02^b^
Other kidney diseases	10 (5.0)	4 (3.4)	6 (7.4)	0.32^a^
DM	26 (13.0)	15 (12.6)	11 (13.6)	0.84^a^
Hypertension	57 (28.5)	27 (22.7)	30 (37.0)	0.03^b^
Hydronephrosis				0.41^b^
Grade 0–2	175 (87.5)	106 (89.1)	69 (85.2)	
Grade 3–4	25 (12.5)	13 (10.9)	12 (14.8)	
Operation				
Stone number				<0.001^b^
Single	142 (71.0)	97 (81.5)	45 (55.6)	
Multiple	58 (29.0)	22 (18.5)	36 (44.4)	
Stone composition				0.45^a^
Calcium oxalate	44 (22.0)	28 (23.5)	16 (19.8)	
Calcium phosphate	10 (5.0)	8 (6.7)	2 (2.5)	
Uric acid and magnesium ammonium phosphate	8 (4.0)	4 (3.4)	4 (4.9)	
Complex	138 (69.0)	79 (66.4)	59 (72.8)	
CT scan (HU)				<0.001^b^
≤1,000	143 (71.5)	97 (81.5)	46 (56.8)	
>1,000	57 (28.5)	22 (18.5)	35 (43.2)	
Stone location				<0.001^a^
Ureter	80 (40.0)	59 (49.6)	21 (25.9)	
Upper calyx	6 (3.0)	3 (2.5)	3 (3.7)	
Median/lower calyx	65 (32.5)	39 (32.8)	26 (32.1)	
Multiple calyx	7 (3.5)	1 (0.8)	6 (7.4)	
Renal pelvis	31 (15.5)	17 (14.3)	14 (17.3)	
Staghorn and full-staghorn	11 (5.5)	0 (0.0)	11 (13.6)	

^a^ Fisher's exact test.

^b^ Chi-square test.

^c^ Wilcoxon two-sample test.

BMI, body mass index; DM, diabetes mellitus; HU, Hounsfield unit.

### Associations between stone size, location, and SFRs

Table 2 shows the univariate and multivariate analyses of outcomes between the two groups. Overall, total one-session SFR and one-month SFR were significantly associated with stone size (both *p* < 0.001) and stone location (both *p* < 0.001). Regarding stone size, stones in the upper calyx and renal pelvis had 100% one-session SFR in both groups, however, staghorn and full-staghorn stones in the size > 2 cm group had low one-session SFRs of 45%. One-session SFR and one-month SFRs were significantly associated with stone location in both groups (*p* = 0.04 for stone <2 cm and *p* = 0.01 for stone >2 cm).

**Table 2:** Associations between stone size, location, SFRs, and complication.

Outcome	Total (*n* = 200)	Stone size (cm)		*p*-value	Multivariable^a^	*p*-value	*S*-value
		<2 (*n* = 119)	≥2 (*n* = 81)		aOR (≥2 vs <2)		
One session SFR							
Total	177/200 (88.5)	114/119 (95.8)	63/81 (77.8)	<0.001^b^	0.22 (0.07–0.67)	0.008	6.97
Ureter	78/80 (97.5)	59/59 (100.0)	19/21 (90.5)	0.07^a^	–		
Upper calyx	6/6 (100.0)	3/3 (100.0)	3/3 (100.0)	–	–		
Median/lower calyx	52/65 (80.0)	34/39 (87.2)	18/26 (69.2)	0.08^b^	0.29 (0.08–1.08)	0.064	4.06
Multiple calyx	5/7 (71.4)	1/1 (100.0)	4/6 (66.7)	1.00^a^	–		
Renal pelvis	31/31 (100.0)	17/17 (100.0)	14/14 (100.0)	–	–		
Staghorn and full-staghorn	5/11 (45.5)	–	5/11 (45.5)	–	–		
*p-value*	<0.001^a^	0.04^a^	0.01^a^				
One-month SFR							
Total	179/200 (89.5)	114/119 (95.8)	65/81 (80.3)	<0.001^b^	0.27 (0.09–0.83)	0.022	5.64
Ureter	78/80 (97.5)	59/59 (100.0)	19/21 (90.5)	0.07^a^	–		
Upper calyx	6/6 (100.0)	3/3 (100.0)	3/3 (100.0)	–	–		
Median/lower calyx	54/65 (83.1)	34/39 (87.2)	20/26 (76.9)	0.32^a^	0.43 (0.11–1.66)	0.22	2.18
Multiple calyx	5/7 (71.4)	1/1 (100.0)	4/6 (66.7)	1.00^a^	–		
Renal pelvis	31/31 (100.0)	17/17 (100.0)	14/14 (100.0)	–	–		
Staghorn and full-staghorn	5/11 (45.5)	–	5/11 (45.5)	–	–		
*p-value*	<0.001^a^	0.04^a^	0.01^a^				
Complication^d^							
Total	5/200 (2.5)	1/119 (0.8)	4/81 (4.9)	0.16^a^	2.62 (0.23–29.32)	0.43	1.20
Ureter	0/80 (0.0)	0/59 (0.0)	0/21 (0.0)	–	–		
Upper calyx	0/6 (0.0)	0/3 (0.0)	0/3 (0.0)	–	–		
Median/lower calyx	1/65 (1.54)	1/39 (2.6)	0/26 (0.0)	1.00^a^	–		
Multiple calyx	0/7 (0.0)	0/1 (0.0)	0/6 (0.0)	–	–		
Renal pelvis	3/31 (9.7)	0/17 (0.0)	3/14 (21.4)	0.08^a^	–		
Staghorn and full-staghorn	1/11 (9.1)	0/0	1/11 (9.1)	–	–		
*p-value*	0.04^a^	0.50^a^	0.04^a^				

SFR, stone-free rate.

^a^ Fisher's exact test.

^b^ Chi-square test.

^c^ Adjusting for stone history, hypertension, stone number, and Hounsfield unit level.

^d^ One patient with a stone size of 1.6 cm had a ureter fissure; four patients with a stone size >2 cm (range 3∼8 cm) had complications (1 with antibiotic medication, 3 with bleeding).

Postoperative complications occurred in only 5 patients. One patient with a 1.6 cm stone located in the middle/lower calyx developed a ureteral fissure, likely due to a thin ureter and the placement of the ureteral access sheath. Among the four patients with a stone size > 2 cm who experienced postoperative complications, one developed a urinary tract infection that required intravenous antibiotic treatment (Clavien-Dindo Grade II), and three experienced gross haematuria without hemodynamic instability. The haematuria resolved spontaneously with conservative management and did not require transfusion or additional interventions (Clavien-Dindo Grade I). The total complication rate was 0.8% for stones <2 cm and 4.9% for stones >2 cm (*p* = 0.16). The complication rate was significantly associated with stone location when stones were >2 cm (*p* = 0.04). Stones located in the renal pelvis had the highest percentage of complications (21.4%), followed by the location of staghorn and full-staghorn stones (9.1%).

After adjusting for history of stones, hypertension, stone number, and Hounsfield unit level, patients with stone size ≥ 2 cm had significantly lower odds of total one-session SFR (aOR: 0.22, 95% CI: 0.07–0.67, *p* = 0.008, *S*-value = 6.97) and one-month SFR (aOR: 0.27, 95% CI: 0.09–0.83, *p* = 0.022, S-value = 1.20) than those with stone size < 2 cm. Stone location was not associated with complications (aOR: 0.16, 95% CI: 0.23–23.32, *p* = 0.43, *S*-value = 1.20).

## Discussion

The present study revealed the high safety and efficacy of SFUI. For stones <2 cm, the total one-session SFR was 95.8% with a complication rate of 0.8%; for stones ≥2 cm, the total one-session SFR was 77.8% with a low complication rate of 4.9%. The low complication rate benefits the fast recovery of patients with large urinary tract calculi.

FURL treatment was reported to have a one-session SFR of 76%-90% for stones ≤2 cm with a complication rate of 6%-7% (2–7, 23, 24), and the SFR decreases to ∼60% for stones of 2–3 cm (25, 26). PCNL was reported to have SFRs higher than 85% with complication rates of 7%-12% for stones <2 cm, and SFRs of 76%-89% with a high complication rate of 10%-25% for stones >2 cm (4–7, 13, 14, 25, 27). The high complication rate of PCNL compared to FURL also leads to significantly longer hospital stays (4, 6, 7). Chen et al. (18) showed that SFUI has a shorter mean operative time, higher stone-free rate, and lower complication rate compared to FURL in cleaning kidney stones <2 cm, while no significant differences were found in postoperative hospital stays between SFUI and FURL (18). Another study showed that SFUI displayed shorter postoperative hospitalization and lower complication rate, including fewer patients needing pain medication and less bleeding compared to mini-invasive PCNL in treating kidney stones of 2–3 cm, while no significant differences were found in stone-free rates and mean operative times between SFUI and PCNL (28). Overall, SFUI displays a better one-session SFR than FURL for stones >2 cm and lower complication rates than PCNL, supporting a favorable option for fast recovery in patients with upper urinary tract calculi of larger size. The intelligence system automatically searched and broke large stones into tiny fragments for direct expulsion. The ability to break and immediately expel stone fragments is the main advantage of the SFUI system, making it markedly different from conventional FURL. In our study, all procedures were performed by a single experienced endourologist, which likely minimized variability related to operator experience. However, previous research has indicated that outcomes may still be influenced by the learning curve, particularly in mastering suction control and sheath positioning (29). Future multicenter studies involving multiple operators are warranted to better quantify the learning curve and its potential impact on clinical outcomes.

Results of the present study showed that SFR is lower in medial/low calyx compared to other locations, and it decreases as stone size increases. Two retrospective studies with small cohorts reported one-session SFRs of 75%–90% with retrograde ureteroscopy and ∼90% with PCNL for lower calyceal stones of 1–2 cm (23, 24). The priority for treating lower calyceal stones is PCNL, while stones <1.5 cm have a better chance for better SFRs with ureteroscopy (30). Meanwhile, staghorn stones are usually large and occupy a large proportion of the renal pelvis. PCNL combined with URL for residual stone cleaning is an option for treating staghorn stones with good final SFR (31, 32). However, the reported one-session SFR with a single PCNL treatment is 55%–60% for staghorn stones, with a high complication rate of ∼30% (33–35,36,37). Regarding the high complication rate of PCNL in treating staghorn stones, SFUI with multiple procedures may be an appropriate alternative consideration.

The SFUI system uses a special ureteral sheath with a pressure monitor on the original flexible scope. The price of this sheath is similar to that of the commonly used flexible ureteroscope sheath without a pressure monitor, making it highly economical and cost-effective. Although special surgical equipment for SFUI is required, the price is not high. Most medical institutions can afford it. Considering that most urinary calculi can be removed without major complications, SFUI is a good choice for both patients and hospitals.

### Limitations

This study has several limitations. First, the absence of a control group undergoing other surgical treatments and the potential influence of unmeasured confounders are major limitations. Second, as a single-center retrospective study, there is a risk of selection bias, potential information bias, and reliance on existing records, which may impact patient recruitment and data analysis, thereby limiting the generalizability of the findings to other institutions or populations. Operation time was not reported. Although the SFUI system can maintain a relatively stable low-pressure state in the kidney, prolonged indwelling of the guide sheath may compress the ureter and cause ureteral injury, particularly in patients with ureteral stenosis, potentially leading to postoperative ureteral strictures. Additionally, longer operative times may increase the risk of other complications, such as lower limb thrombosis. Moreover, stone complexity was not graded using Guy's score due to incomplete anatomical data in some imaging records, which limited our ability to apply the scoring system consistently across all patients and may reduce the granularity of outcome interpretation. Another imitation is the lack of systematically recorded quantitative urine culture data, which may limit the ability to fully assess preoperative urinary infection status. Further prospective studies are highly recommended to specifically address these concerns and provide a more comprehensive analysis, including a direct comparison with PCNL.

## Conclusions

SFUI system is safe and effective for immediate stone-free. The high SFR and very low complication rate benefit fast recovery, especially for patients with large calculi in the middle/lower calyx and renal pelvis.

## Data Availability

The original contributions presented in the study are included in the article/Supplementary Material, further inquiries can be directed to the corresponding author.
